# Supplementary figures and images for: Improving retinal vascular endothelial cell tropism through rational rAAV capsid design
Source: PLoS One. 2023 May 11;18(5):e0285370. doi: 10.1371/journal.pone.0285370 (PMC10174500; doi:10.1371/journal.pone.0285370)

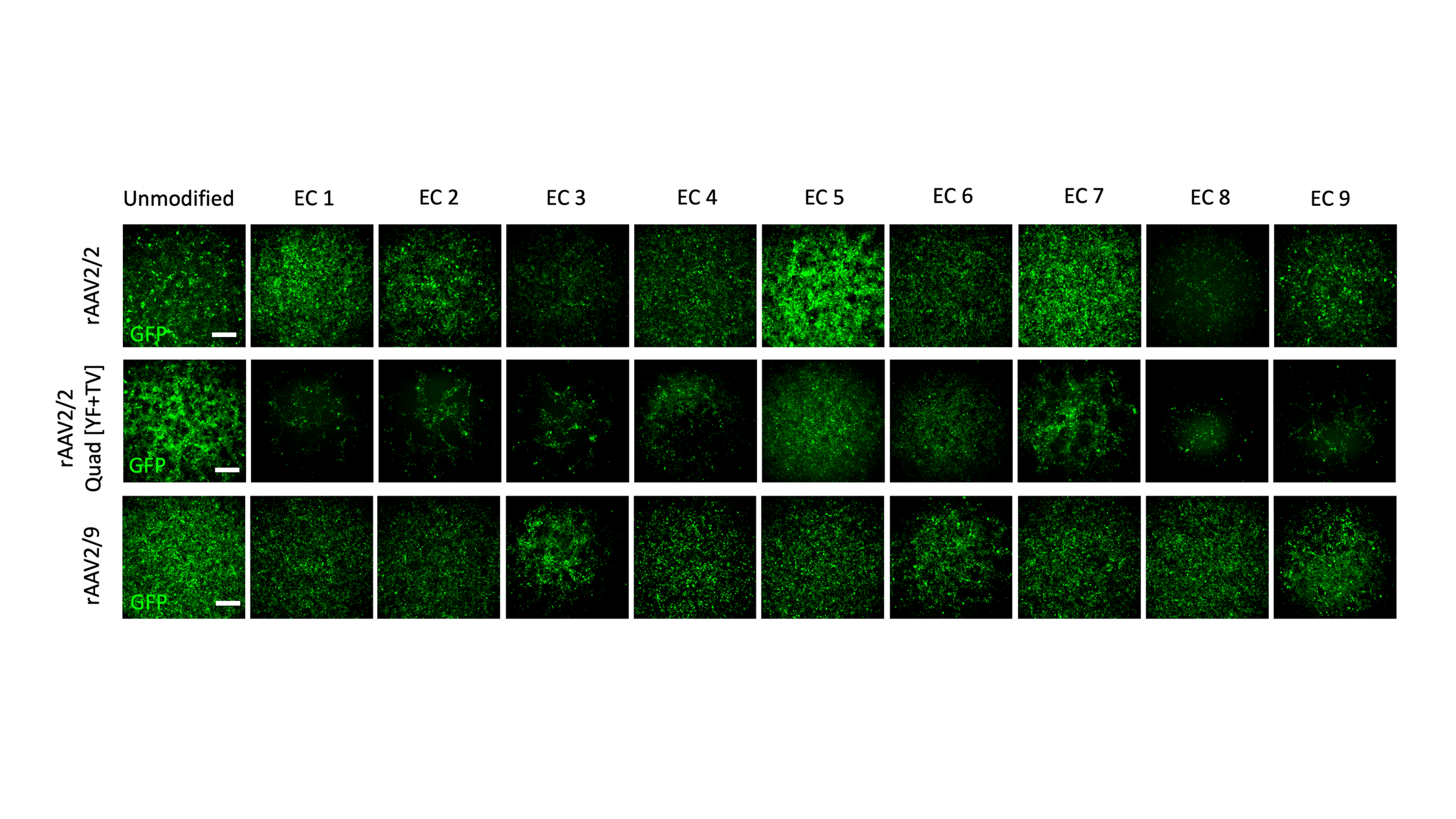

Supplement: S1 Fig — All vectors at 50,000 MOI were introduced to HEK293T cells and observed under a Fluorescence microscope for GFP expression after 48 hours. Scale bar: 150μm. (TIF) [file pone.0285370.s001.tif]

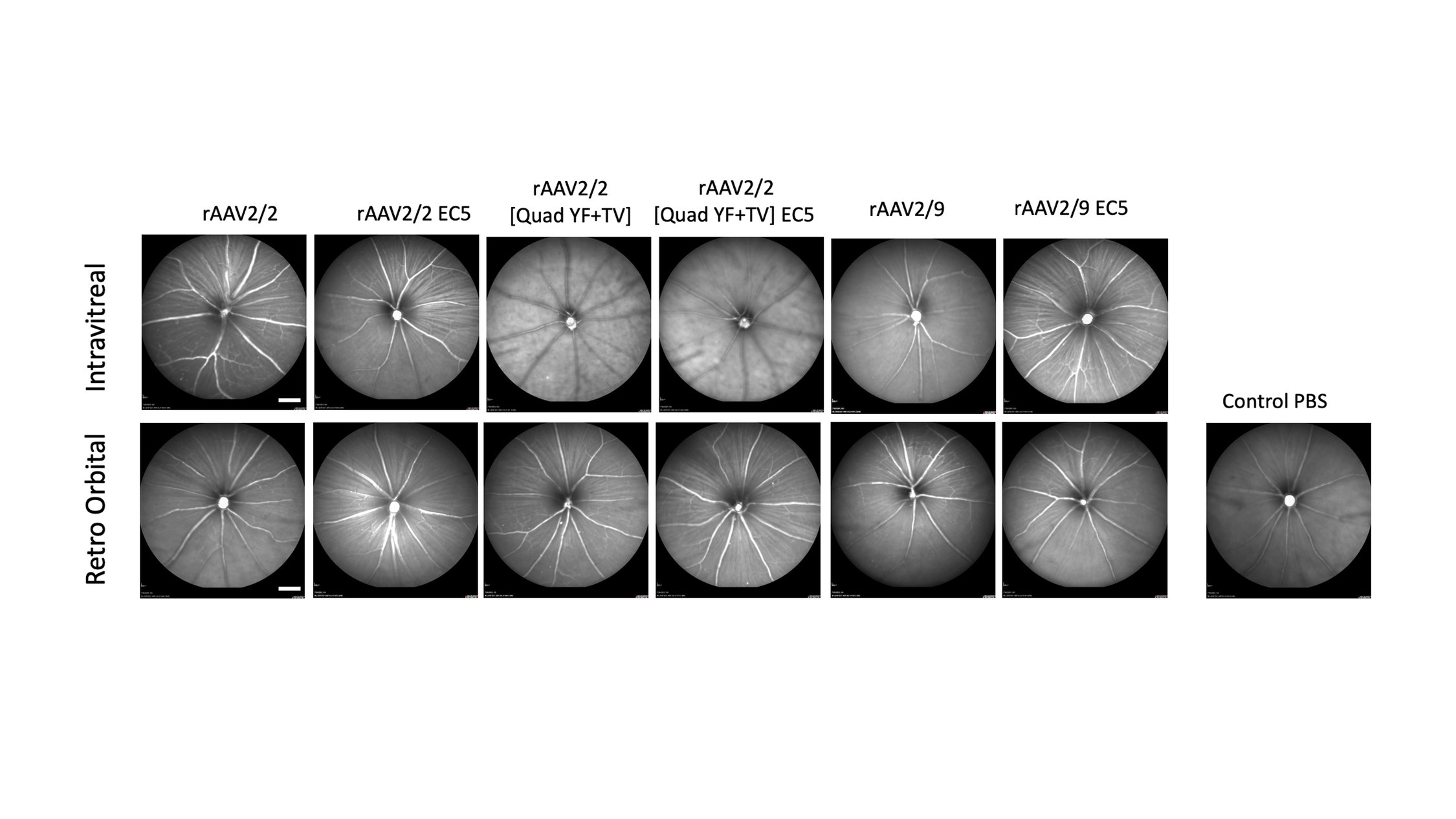

Supplement: S2 Fig — Near-infrared (820nm) reflectance imaging was performed using Confocal scanning laser ophthalmoscopy (cSLO) four months post-injection, and No evidence of ocular damage was observed. The above figure represents the cSLO images of mice eyes that received Intravitreal and Retro Orbital injections (2ul each) of all vectors and control (PBS). Scale bar: 800μm. (TIF) [file pone.0285370.s002.tif]
